# Airway branching morphogenesis in three dimensional culture

**DOI:** 10.1186/1465-9921-11-162

**Published:** 2010-11-25

**Authors:** Sigrídur R Franzdóttir, Ivar T Axelsson, Ari J Arason, Ólafur Baldursson, Thorarinn Gudjonsson, Magnus K Magnusson

**Affiliations:** 1 Stem Cell Research Unit, Biomedical Center, School of Health Sciences, University of Iceland, Reykjavik, Iceland; 2 Department of Pharmacology & Toxicology, School of Health Sciences, University of Iceland, Reykjavik, Iceland; 3 Department of Laboratory Hematology, Landspitali University Hospital, Reykjavik, Iceland; 4 Department of Pulmonary Medicine, Landspitali University Hospital, Reykjavik, Iceland

## Abstract

**Background:**

Lungs develop from the fetal digestive tract where epithelium invades the vascular rich stroma in a process called branching morphogenesis. In organogenesis, endothelial cells have been shown to be important for morphogenesis and the maintenance of organ structure. The aim of this study was to recapitulate human lung morphogenesis *in vitro *by establishing a three dimensional (3D) co-culture model where lung epithelial cells were cultured in endothelial-rich stroma.

**Methods:**

We used a human bronchial epithelial cell line (VA10) recently developed in our laboratory. This cell line cell line maintains a predominant basal cell phenotype, expressing p63 and other basal markers such as cytokeratin-5 and -14. Here, we cultured VA10 with human umbilical vein endothelial cells (HUVECs), to mimic the close interaction between these cell types during lung development. Morphogenesis and differentiation was monitored by phase contrast microscopy, immunostainings and confocal imaging.

**Results:**

We found that in co-culture with endothelial cells, the VA10 cells generated bronchioalveolar like structures, suggesting that lung epithelial branching is facilitated by the presence of endothelial cells. The VA10 derived epithelial structures display various complex patterns of branching and show partial alveolar type-II differentiation with pro-Surfactant-C expression. The epithelial origin of the branching VA10 colonies was confirmed by immunostaining. These bronchioalveolar-like structures were polarized with respect to integrin expression at the cell-matrix interface. The endothelial-induced branching was mediated by soluble factors. Furthermore, fibroblast growth factor receptor-2 (FGFR-2) and sprouty-2 were expressed at the growing tips of the branching structures and the branching was inhibited by the FGFR-small molecule inhibitor SU5402.

**Discussion:**

In this study we show that a human lung epithelial cell line can be induced by endothelial cells to form branching bronchioalveolar-like structures in 3-D culture. This novel model of human airway morphogenesis can be used to study critical events in human lung development and suggests a supportive role for the endothelium in promoting branching of airway epithelium.

## Introduction

Lung development and critical aspects of pulmonary epithelial differentiation is mostly studied through the use of animal models[1]. Due to a lack of good experimental *in vitro *models, much less is known about development and stem cell biology in human lungs. While many different human airway epithelial cell lines capture the phenotypic traits of the proximal airways such as trachea and large bronchi [2-4], there is lack of cell lines that mimic normal histological features of the lung, such as branching morphogenesis of the distal airways. Furthermore, there are inherent differences in the cellular composition of the airway epithelium between rodents and humans. In the rodent, basal cells, candidate airway epithelial stem cells, are confined to the trachea, while in the human lung basal cells are present throughout the upper airways, and all the way down to small bronchioles [5-7]. This supports the importance of generating models of human airway development and differentiation to study the cell biology of the human lung including epithelial stromal interactions and branching morphogenesis.

Although many human airway epithelial cell lines have been established, most of them have not been defined with respect to their cellular origin and lack critical characterization in terms of expression of differentiation markers[2]. The most cited airway epithelial cell line, A549, is derived from a human bronchioalveolar carcinoma [8]. Despite its origin in malignant tissue it has been widely used to study lung biology. The human bronchial cell lines 16HBE14o-, Calu-3, and BEAS-2B have been successfully applied to study drug transport, metabolism, and drug delivery due to their ability to form tight junctions (TJ) [2]. The Calu-3 [3] and 16HBE14o [4]cell lines have been identified as the most differentiated cell lines available and have been used to study bronchial epithelial integrity including barrier function and the activity of tight junctions complexes [2].

In order to mimic the airway epithelial lining, primary human bronchial epithelial cells have been studied under various conditions. When primary human epithelial cells are cultured at the air-liquid interface using serum containing differentiation media, they undergo terminal squamous differentiation instead of forming a pseudostratified polarized and ciliated epithelial layer [9]. However, under the same conditions fibroblasts and fibroblast secretions have been shown to stimulate the formation of a pseudostratified ciliated epithelium [10]. This highlights the importance of the bi-directional communication between the epithelial and stromal cellular compartments. Recently, human alveolar type II cells were shown to form cysts in 3D culture through a novel mechanism of epithelial morphogenesis relying on aggregation and rearrangement [11]. In this model of terminal airway cyst formation using Matrigel based 3-D culture conditions, no branching morphogenesis occurred.

Most studies on epithelial-mesenchymal interactions have focused on fibroblasts and components of the extracellular matrix. Less is known about the role of the vascular endothelium and its interaction with epithelial cells. Mouse culture models, e.g. lung tissue explants have though shown a critical role for the interaction between the vascular and epithelial compartments during lung development [12]. Interestingly, it has been shown that endothelium-derived factors are necessary for distal lung morphogenesis and function in mice, including the formation of alveoli [13] and the maintenance of alveolar integrity [14]. Furthermore, *in vitro *models suggest that endothelial cells support airway epithelial tight junction formation [15]. Recent data from other organs such as the liver, pancreas, brain and bone marrow indicate that organ specific endothelial cells are of major importance for fate control of stem cells as well as for organogenesis and tissue maintenance [16]. Better understanding of the heterotypic crosstalk between the epithelium and the surrounding stroma is important to understand such important events as lung development, lung carcinogenesis and repair of the airway epithelium following injury.

In this paper, we describe a 3D epithelial culture model using a recently described human basal-like airway epithelial cell line (VA10) cultured in reconstituted basement membrane (rBM) [17]. VA10 cells form a pseudostratified layer in air-liquid culture and have been used to study airway epithelial defense mechanisms, including tight junction function and the production of antimicrobial peptides [17-19]. In contrast, when cultured in rBM matrix, a condition more favorable for distal lung morphogenesis, VA10 cells form spheres with a clear apical-basal polarity and tight intercellular junctions as shown by the expression of β4-Integrin on the outer (basal) surface, and claudin-1 laterally, but lack branching morphogenesis [17]. VA10 has a phenotype similar to human airway epithelial basal cells, including the expression of p63 and cytokeratin 14 [17]. Airway epithelial basal cells both self-renew and generate luminal daughter-cells in a sphere-forming assay, and have thus been suggested to be candidate airway epithelial stem cells [6]. This suggests that VA10 might be an ideal cell line to use in developmental and morphogenesis studies of human lung differentiation. To mimic heterotypic cellular interactions we have co-cultured the VA10 cells with vascular endothelial cells in a 3D environment, and under these conditions we are able to show marked branching morphogenesis and a differentiation profile of the epithelial cells towards an alveolar epithelial phenotype. Such branching morphogenesis is novel in a human *in vitro *model. An *in vitro *cellular model of lung development and differentiation can serve as an important platform to study critical events, such as cellular interaction and cell signaling in lung morphogenesis. Furthermore, our data support a critical interaction between the vascular and epithelial compartments during epithelial branching morphogenesis and differentiation.

## Materials and methods

### Cell culture

The bronchial epithelial cell line VA10 was previously established by retroviral transduction of primary bronchial epithelial cells with E6 and E7 viral oncogenes ([19]). The cells were cultured in bronchial epithelial growth medium, BEGM (Lonza, Walkersville, MD) supplemented with 50 IU/ml penicillin and 50 μg/ml streptomycin (Gibco, Burlington, Canada).

The human lung adenocarcinoma derived alveolar epithelial cell line A549 (American Type Culture Collection, Rockville MA) was cultured in DMEM-Ham's-F12 basal medium supplemented with 10% fetal bovine serum (FBS), 50 IU/ml penicillin and 50 μg/ml streptomycin (Gibco).

Primary human umbilical vein endothelial cells were cultured on T75 tissue culture flasks (Becton Dickinson) on endothelial medium EGM-2 supplemented with 50 UI/ml penicillin, 50 μg/ml streptomycin (Gibco) and 30% FBS for first passage cells or 5-10% FBS after first passage. Endothelial cells were only used up to passage 8.

### Three-dimensional culture

For 3D culture experiments growth factor reduced reconstituted basement membrane (Matrigel, BD Biosciences, Bedford, MA) was used. Cells were seeded into 300 μl of Matrigel in its liquid state, plated into 24-well culture dishes and allowed to gelatinize at 37°C for 30 minutes before adding 1 ml of culture medium. For co-culture experiments 2.5 × 10^6 ^HUVEC cells and 500-1000 VA10 (or A549) cells were seeded into the Matrigel to ensure clonal growth and reduce cell aggregation of epithelial cells. No clumping of cells was observed before seeding. In this experimental setup the endothelial cells remain non-proliferating while marked proliferation is seen in the epithelial cells. For direct staining of branching structures in Matrigel we used 8-well chamber slides with 100 μl Matrigel, 8.3 × 10^5 ^HUVEC cells and 333 VA10 cells. For transwell (TW) experiments, HUVEC cells were seeded onto 0,4 μm polyester filter inserts (Corning, MA) and allowed to reach 70% confluence before onset of experiment. 333 VA10 cells were seeded into 100 μl Matrigel in 24-well plates and allowed to gelatinize before the TW filters and culture medium were added to the wells. For FGFR inhibition, the pan-FGFR inhibitor SU5402 was diluted to the indicated concentration from a 100 mM DMSO stock solution in the culture medium. The medium was replaced three times a week. The corresponding concentration of DMSO was used in the control medium.

### Immunohistochemistry

Immunofluorescent staining of 3D gels was carried out in the culture chamber as previously described [20]. The gels were washed twice with PBS and fixed in methanol (10 minutes at -20°C). For some primary antibodies we used double acetone fixation with ice cold acetone for ten minutes at 4°C followed by drying and repeated acetone treatment. After fixation the gels were washed three times for 15 minutes at room temperature with 100 mM Glycine in PBS, and blocked with 10% goat serum in IF-buffer (0.2% Triton X-100; 0.1% BSA and 0.05% Tween-20 in PBS). To block unspecific binding to the mouse-derived rBM gel, the IF-buffer was supplemented with 1% goat anti mouse immunoglobulin G for 20 minutes. Primary antibodies were incubated overnight at 4°C, followed by three 25 minute washes in 10% Goat Serum in PBS. The secondary antibodies were incubated for 2 hours at RT or over night at 4°C. After PBS rinsing, nuclear staining was performed with TO-PRO-3(r) (Invitrogen, Carlsbad, CA) for 30 minutes followed by 3 × 15 minute washes. The chambers were removed after staining and the samples were embedded in Fluoromount-G (Southern Biotech, Birmingham, AL) for microscopic analysis.

For freeze sections, the gels were flash-frozen in n-hexan and transferred to -80°C. The gels were mounted in Tissue-Tek(r) O.C.T. compound (Sakura Finetek, Zoeterwoude, Netherlands) and sectioned in a cryostat (5-20 μm sections). The samples were dried and then fixed and stained as above, with antibody incubations for 30 minutes at RT.

The following antibodies were used: Rabbit polyclonal antibodies to pro-SPC (AB3786) (Abcam, Cambridge, MA). Mouse monoclonal antibodies to: CD31 (JC/70A), Cytokeratin 17 (E3) (DAKO, Glostrup, Denmark); Cytokeratin 14 (LL02), FGFR2 (Abcam, Cambridge, MA); β4-Integrin (3E1) (Chemicon, Temecula, CA); p63 (7JUL), TTF-1 (SPT24) (Novocastra Laboratories, Newcastle upon Tyne, UK); E-Cadherin (HECD1) (Zymed, South San Francisco, CA).

Isotype specific secondary antibody conjugates Alexa fluor(r) (Alexa fluor, 488 (green), 546 (red), Invitrogen) were used for immunofluorescence experiments with TO-PRO-3(r) (Invitrogen) for nucleic acid staining. Antibody incubations were carried out for 30 minutes at room temperature with secondary antibodies and nucleic stain in darkness. Specimens were rinsed twice for 5 minutes at room temperature between antibody and nucleic stain incubations. For maximum preservation of the fluorescent signal from the samples after staining, specimens were mounted using Fluoromount-G (Southern Biotech, Birmingham, AL) and images visualized with confocal microscope.

### Confocal microscopy

Immunofluorescence was visualized and captured using laser scanning Zeiss LSM 5 Pascal Confocal Microscope (Carl Zeiss AG, Munich, Germany). Bright-field and phase-contrast images of Matrigel cultures were captured using a Leica DFC320 digital camera attached to a Leitz Fluovert microscope (Wetzlar, Germany)

## Results

### Endothelial cells stimulate branching morphogenesis of airway epithelial cells

**Figure 1:**
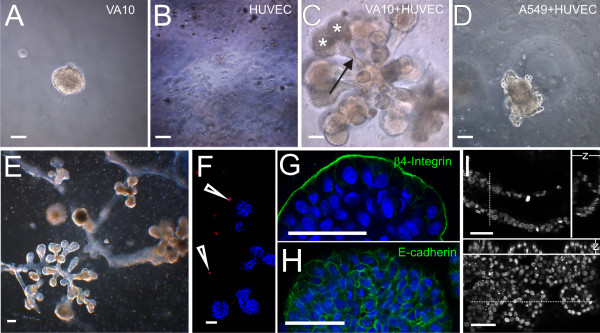
**Endothelial cells induce branching in colonies of VA10 epithelial cells in reconstituted basement membrane matrix**. **A-E**. Phase contrast images of cells cultured in rBM. **A**. VA10 cells form spherical colonies when cultured in rBM. **B**. HUVEC cells cultured in rBM do not form colonies but occur as single non-proliferative cells. **C**. Complex epithelial branching structures form when VA10 cells are co-cultured with endothelial cells. Secondary bifurcations (asterisks) are seen at the ends of elongated branches (arrow). **D**. A549 cells form colonies that do not branch when co-cultured with HUVEC. **E**. Co-culture of VA10 and HUVEC cells, showing extensive branching network formation after 19 days in culture. **F-I**. Confocal images of branching structures. **F**. Cryosection stained with a nuclear marker (TO-PRO-3, blue) and CD31 (red) to label endothelial cells, see arrowheads. **G, H**. Details of terminal buds, TO-PRO-3, blue. **G**. β4-Integrin expression (green) at the interphase between the structures and the matrix. **H**. E-Cadherin (green) outlines the epithelial cell junctions in branching structures. Scale bars 100 μm (A-E)/50 μm (F-I) **I**. Confocal sections and orthogonal sections showing gaps in the nuclear pattern (TO-PRO-3, white) in a tube (upper half) and terminal buds (lower half). Dotted lines indicate the location of the orthogonal sections (-z-).

In the light of recent data from various organs demonstrating the importance of vascular endothelium in stem cell niche and organogenesis [21-23], and due to the fact that bronchioles and alveoli are adjacent to the vascular endothelium *in vivo*, we hypothesized that vascular endothelium might induce a distal airway phenotype in bronchial epithelial cells. We first tested the phenotypic behavior of VA-10 when cultured alone in rBM 3D matrix, a condition favorable for distal lung morphogenesis. When cultured in 3D-rBM, the VA10 cells formed spherical colonies without branching (figure 1A). We have previously shown these spherical colonies to have a clear apical-basal polarity and tight intercellular junctions as shown by the expression of β4-Integrin on the outer (basal) surface, and claudin-1 laterally[17]. Human umbilical vein endothelial cells (HUVEC) alone under these culture conditions remained non-proliferative but viable for over 4 weeks and did not form any structures (figure 1B). The viability and metabolic activity of HUVECs was verified through the uptake of acetylated-low-density lipoprotein (data not shown). To test the potential heterotypic interaction between pulmonary epithelial cells and endothelial cells, we designed a co-culture assay mixing VA10 airway epithelial cells and endothelial cells. When HUVECs were seeded together with the VA10 cells into rBM, the HUVECs remained quiescent but a striking proliferative and morphogenic effect was seen in colonies derived from VA10 cells. They formed complex branching structures reminiscent of bronchioalveolar units of the developing lung (figure 1C), with short, thin branches ending in alveolar-like buds, or cleaving at the tips to form secondary branches (figure 1C, E, G). In general, most forms of structures seen are densely packed with nuclei (figure 1G &1H). However, minor hollow spaces/cavities are seen in a subset of the branching colonies (50% of budding colonies, 40% of early branching and 31% of complex branching colonies examined, n = 43). The cavities are generally small and few (1-2 per colony), but in a few structures a more air-space like pattern is observed (Figure 1I). The cells show epithelial characteristics such as polarity towards the rBM indicated by the expression of β4-integrin on the basal surface (external surface of the structures, figure 1G), and E-cadherin expression at cell-cell contacts (figure 1H). The endothelial marker CD31 was used to identify the position of HUVEC cells (figure 1F). Individual endothelial cells were seen distributed throughout the gel, while no staining was detected within the branching structures, confirming that the HUVECs do not contribute directly to the structures.

**Figure 2:**
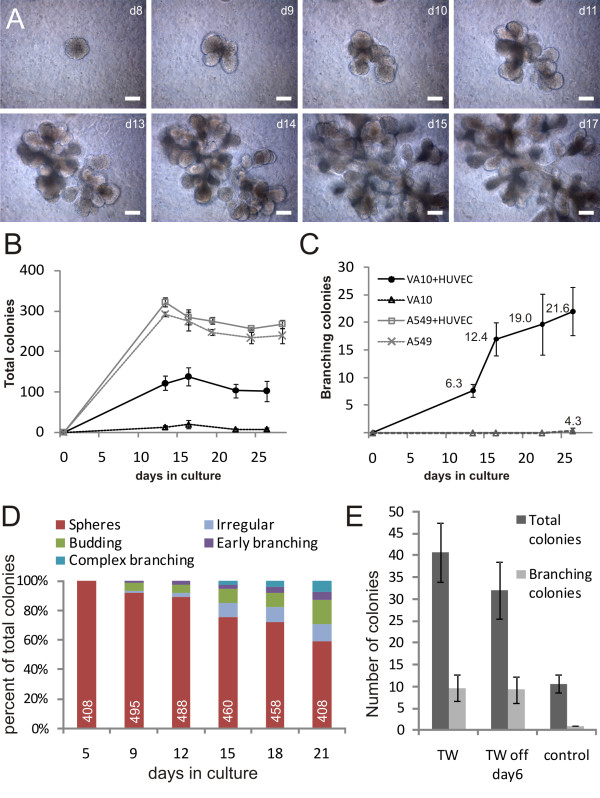
**Endothelial cells induce various branching phenotypes in a time-dependent fashion through soluble factors**. **A**. Time series of VA10/HUVEC co-culture in Matrigel. Days after seeding are indicated. A colony undergoing branching morphogenesis is shown during days 8-17 after seeding. The colonies can be categorized as follows: Day 8 spherical; day 9 budding; days 10-12 early branching; days 13-17 complex branching. Scale bars 100 μm. **B**. The number of colonies per well over time in A549 rBM culture (crosses), VA10 culture (triangles), and co-culture of HUVEC cells with A549 (empty boxes) or VA10 cell (solid circles). **C**. The number of branching colonies over time in co-culture of VA10 and HUVEC and VA10 cells cultured alone. The percentage of total colonies is shown above each point. Error bars indicate standard deviation. **D**. The proportion of the different colony forms at several time-points. Irregular colonies do not show any sign of regular branching but cannot be categorized as spherical. The total number of colonies is indicated at the base of each column. **E**. Culture of endothelial cells on transwell (TW) filters suspended above rBM gels containing VA10 cells. Total and branching epithelial colonies are indicated. Error bars indicate standard deviation. TW: HUVEC kept on filters throughout experiment. TW off day 6: Transwells were removed on day 6. Control: Negative control without HUVEC in TW.

To further investigate the process of branching morphogenesis in culture, we analyzed a time-lapse image series of VA10 cells in co-culture with endothelial cells and quantitated the effects of co-culture with endothelial cells both on VA10 cells and A549 (figure 2). In general the structures formed from single colonies of VA10 cells. No clumping of VA10 cells was observed before seeding and a similar pattern of branching morphogenesis was seen in co-cultures where the VA10 were passed through a single cell filter prior to seeding [see Additional file 1]. Figure 2A details the events of branching morphogenesis in the model. Minor clefts can be seen in the round sphere at day 8. At day 9 these clefts have grown larger as the initial branches have started budding out from the colony (budding). The branches extend and generate secondary (early branching) and even tertiary branches, thus showing complex epithelial branching (complex branching). Each branch terminates in an alveolar-like bud. The HUVECs not only induced branching in VA10 cells (figure 2C), but also stimulated the total number of spherical colonies (figure 2B). The earliest branching colonies were seen after 9 days in culture, but a rapid increase in their number was seen after 13 days (figure 2C), with 6,3% of colonies branching at day 13 and 21.6% at day 26. Branching colonies are also occasionally observed after prolonged culture periods of VA10 cells without endothelial cells being present, however these branching colonies are less than 1% of the total. Figure 2D enumerates the proportion of each branching phenotype, spherical, budding, early and late branching and irregular (irregularly shaped structures that do not conform to the other categories). As seen, after three weeks in culture, over 40% of the colonies showed some branching phenotype. Even though the number of spherical colonies still outnumbered branching colonies, the branching colonies were much larger.

We also tested if the widely used human alveolar epithelial cell line A549 could generate branching colonies in rBM. When cultured alone, A549 cells formed irregular colonies and no phenotypic changes occurred in co-culture with endothelial cells, indicating lack of ability to generate branching structures (figure 1D). Furthermore, the endothelial cells did not increase the number of colonies or degree of branching (figure 2B).

To study the nature of the endothelial-induced branching, we set up a transwell co-culture system where the endothelial cells were cultured on top of the filter situated above the 3D gels containing VA10 cells. When endothelial cells were present on the filters a marked stimulation was seen in the total number of colonies seen in the 3D culture, and again the branching phenotype was dependent upon their presence (Figure 2E). This suggests soluble, endothelial derived factors as the mediators of inducing the branching morphogenic phenotype. We also removed the endothelial filters at a time before the earliest signs of branching (day 6), and in this setup, the VA10 cells still showed marked branching suggesting the endothelial cells are inducing a phenotypic switch at an early stage in the 3D colonies (Figure 2E).

### VA10-derived bronchioalveolar-like structures show partial alveolar type II phenotype

**Figure 3:**
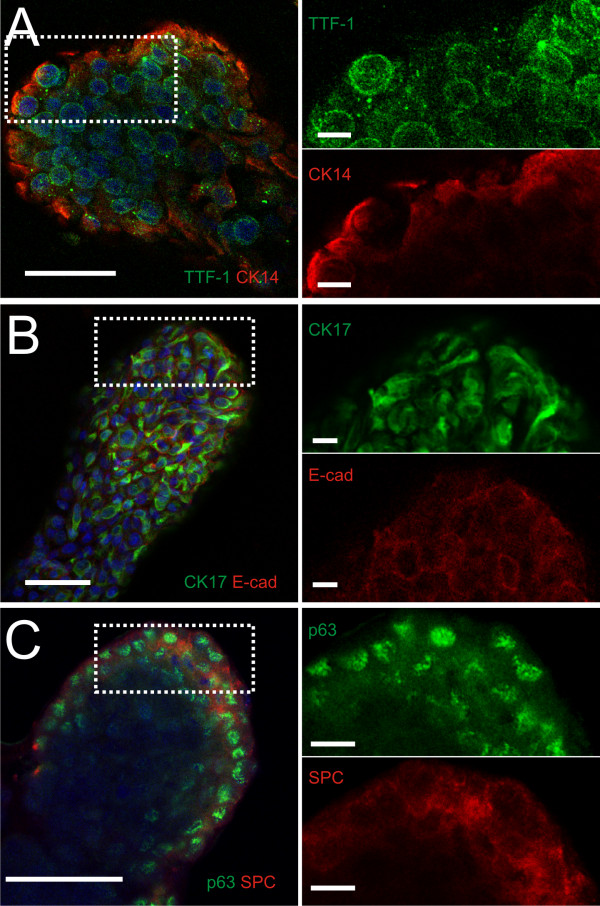
**The branching epithelium expresses basal and bronchial epithelial markers**. Confocal sections of immunofluorescently labeled structures in intact Matrigel. Nuclear staining with TO-PRO-3 is shown in blue in all panels. The boxed areas are shown in detail to the right. **A**. TTF-1 (green) protein is seen in all nuclei with weaker expression towards the core of the structure. CK14 expression (red) is limited to the outermost cells of each structure. **B**. CK17 in green, E-Cadherin in red. **C**. p63 is expressed in all cells (green) and pro-SP-C expression (red) is seen throughout the structures, and is most prominent at the outer cell layers. Scale bars 50 μm (left column), 10 μm (right column).

We analyzed the expression of several epithelial cell markers in the complex branching structures formed in the co-culture conditions (figure 3). Thyroid transcription factor 1 (TTF-1) is the earliest known marker of lung epithelial cell commitment during mouse development. TTF-1 expression is observed throughout the branching structure in a nuclear pattern (figure 3A), similar to the expression seen in the terminal respiratory unit of normal lungs [24]. In addition to the TTF-1 lung marker expression, markers of both basal and alveolar type II epithelial cells are co-expressed in the branching structures. Basal cells of the human airway epithelium express the nuclear protein p63, as well as certain cytokeratins, including CK14 and CK17, not expressed by other airway epithelial cells. These factors are all expressed in the branching structures, p63 showing nuclear staining throughout the structure (figure 3C), CK17 in the cytoplasm (shown together with E-cadherin in figure 3B) and CK14 showing strong expression in cells facing the rBM (figure 3A). Surfactant protein-C (SP-C) is normally expressed and secreted by alveolar type-II cells. Cytoplasmic expression of the propeptide (proSP-C) is seen in the branching structures (figure 3C), together with the p63 protein, indicating a partial differentiation of the basal-like cells towards an alveolar type II fate. Thus, the branching VA10 colonies express pulmonary specific genes such as TTF-1 and proSP-C. Despite the partial differentiation towards type-II alveolar cells, the VA10 cells retain basal markers such as CK14/17 and p63. The expression of these proteins in the other epithelial 3D phenotypes (spherical, budding, early branching) is shown in supplement data [Additional file 2]. The expression of CK14 is interesting, showing individual CK14 positive cells interspersed throughout structures at earlier stages, while organizing in a linear fashion at the outer surface in the mature, complex branching structures.

**Figure 4:**
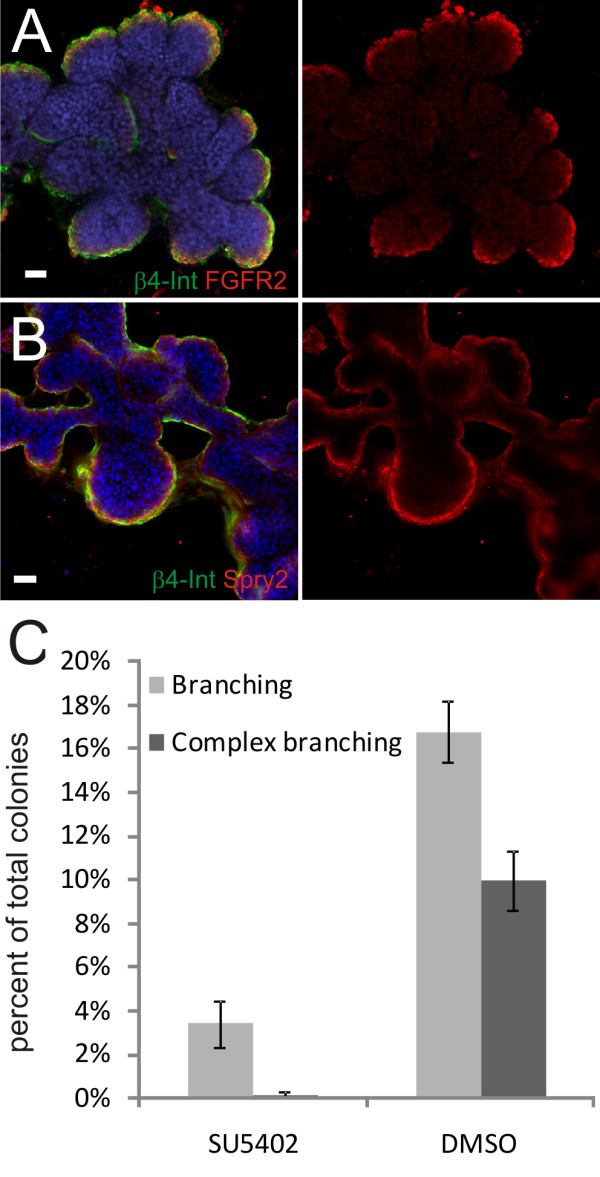
**FGFR2 and Sprouty2 are expressed in the branching epithelium. Inhibition of FGFR signaling inhibits branching**. **A, B**. Confocal sections of isolated structures, nuclear TO-PRO-3 staining is shown in blue, β4-Integrin in green. **A**. FGFR2 (red) is up-regulated at the tips of branches. **B**. Sprouty2 expression lines the branching structures. Scale bars 50 μm. **C**. Inhibition of FGFR signaling. 25 μM SU5402 or DMSO were added to the cultures after 5 days. The graph shows the proportion of branching (light-gray) and complex branching (dark-gray) colonies at day 13. Error bars indicate standard deviation. SU5402 n = 1135; DMSO n = 952 colonies total.

Given the extensive growth and branching seen in the co-culture model, we analyzed the expression of two critical regulators of branching morphogenesis and lung development, FGFR2 and Sprouty-2. FGFR2 is expressed in the branching structures with most pronounced expression at the growing end-buds (figure 4A). Sprouty-2 expression, on the other hand, is more uniform along the whole lining of the structures, lining both the end-buds and the stalks (figure 4B). The expression of these factors is similar to what is seen in animal models of lung development and indicates that the same molecular events are likely to underlie branching morphogenesis in our culture model system as seen *in vivo *in rodent lung models and the Drosophila airway conducting system [25,26]. Given this expression pattern of FGFR2, we used a small molecule non-specific FGFR inhibitor, SU5402 [27] to block FGF signaling. When SU5402 (25 or 50 μM) was present throughout the culture period there was a strong inhibitory effect on colony growth, only 32% of the control colony number was present at 25 μM of SU5402 and very few colonies present at 50 μM [Additional file 3]. No branching was observed in the SU5402 cultures. Instead, the colonies adopted a grape-like phenotype [Additional file 3]. Given the profound inhibitory effect on colony growth, a separate set of experiments was performed where SU5402 (25 μM) or DMSO was added to the cultures at day 5 post seeding, i.e. after the colonies had reached a size over 50 μm. By day 10, a similar number of early-branching colonies was seen in control and SU5402 wells. However, by day 13 four times more branching colonies were seen in the control compared to cultures treated with the FGFR inhibitor (Figure 4C). No complex branching was seen with the inhibitor. Thus, inhibition of FGF receptor has a strong effect on the branching ability of VA10 cells in co-culture with endothelial cells.

## Discussion

*In vitro *models are important supplements to animal models for studying cellular interactions and basic developmental processes, repair and carcinogenesis. In this paper we describe a novel *in vitro *model of branching morphogenesis in the human lung, using an airway epithelial cell line in co-culture with endothelial cells, which captures critical aspects of distal lung morphogenesis. We found that in this system branching morphogenesis is depended on an interaction between airway epithelia and the vascular endothelium.

The stroma is known to play a major role in organogenesis and maintenance of tissue structure and function in epithelial based organs such as the mammary gland [28], prostate[29] and lung[14,30]. As the lung initially develops from the fetal foregut it is highly dependent on crosstalk between the endodermal cells and the underlying stroma. The outgrowth and branching of epithelial cells is stimulated by various stromal-derived growth factors, especially fibroblast growth factors (FGFs) acting through FGF-receptors on the invading epithelial cells [31]. Studies on mouse embryos and fetal lung tissue explants have shown the stroma to play a critical role in the invasion and branching of the airway epithelium during mouse lung development [32,33]; however, these studies have not directly identified the cellular components of the stroma that mediates these effects. Our model using a bronchial-derived epithelial cell line with a basal-like phenotype suggests that endothelial cells could be critical mediators in stimulating epithelial invasion and branching behavior through secretion of soluble factors.

Branching morphogenesis is one of the key developmental processes during lung development. A recent seminal paper studying branching morphogenesis of the mouse lung indicated that this process is based on a pattern of three simple branching modes, repeated in different order throughout lung development. The authors proposed that these simple branching modes were controlled genetically through master regulators [34]. They suggested that the *sprouty *gene family might be among the developmental switches or regulators of this process, specifically *sprouty-2*. In our model, both FGFR2 and Sprouty-2 are highly expressed at the growing buds of the branching structures. In the mouse embryonic lung, Sprouty-2 appears to be dynamically expressed in the peripheral endoderm and down-regulated in the clefts between new branches. Furthermore, over-expression of Sprouty-2 in the peripheral airway epithelium *in vivo *results in diminished branching [25]. The ability of VA10 cells to form bronchioalveolar-like structures in co-culture with endothelial cells opens the possibility to study important aspects of human lung morphogenesis, such as the spatial and temporal function of FGF signaling and Sprouty, in a well controlled *in vitro *system. Furthermore, initial data supports a role for FGFR signaling in our model, although we cannot conclude that the main endothelial derived branching factor is an FGF growth factor given the abundance of FGF in both serum and the growth medium.

In the adult lung, the stroma changes substantially from the proximal conducting part to the distal alveolar part. In the larger bronchi, cartilage tissue supports the epithelial tissue. Fibroblasts, smooth muscle cells, and large vessels are also prominent in the proximal part. In the distal bronchioalveolar zone the cartilage has disappeared and microvessels are prominent, especially surrounding the alveoli. Recent data from various organs such as the liver, pancreas, brain and bone marrow indicate the importance of endothelial cells in fate control of stem cells and for organogenesis and tissue maintenance [16]. Lammert et al. have shown that endothelial cells are important for both pancreas and liver organogenesis [21]. Similarly, endothelial cells were shown to be vital components of the stem cell niche in the nervous system [23], and in hematopoiesis [35]. It is thus becoming evident that endothelial cells are important regulators of stem cells in many organs and a crucial component for cell fate decisions and tissue morphogenesis.

Much less is known about somatic stem cells of the lung compared to many other epithelial organs, such as gut, breast and prostate. One of the reasons is the histological difference between the proximal and distal part of the lung and the cellular complexity found within the airway epithelium. Candidate stem cell types include basal cells, Clara cells and alveolar type II cells[36]. Of these, basal cells are postulated to be the most pluripotent candidate stem cells [6]. Thus, the basal cell characteristics of VA10 make it an interesting candidate for testing stem cell properties. Indeed, this cell line can form a pseudostratified layer with mature TJs under air-liquid interphase conditions [17], suggesting that it differentiates significantly depending on environmental conditions. In addition we show that the VA10 cell line forms branching epithelial structures reminiscent of distal bronchioalveolar-like structures with partial alveolar type II differentiation, yet retaining a basal cell phenotype.

## Conclusions

In summary, we have described a novel cell culture model that captures critical aspects of human airway branching morphogenesis. This model provides a unique tool to study and characterize various processes of human lung development, morphogenesis and to address the heterotypic interaction between epithelial and stromal components during airway differentiation and tubular branching morphogenesis. Furthermore, our results identify endothelial cells as potential inducers of distal airway epithelial differentiation.

## Abbreviations

3D: three dimensional; FBS: fetal bovine serum; FGFR-2: fibroblast growth factor receptor-2; FGFs: fibroblast growth factors; HUVECs: human umbilical vein endothelial cells; proSP-C: pro-Surfactant protein-C; rBM: reconstituted basement membrane; SP-C: Surfactant protein-C; TJ: tight junctions; TTF-1: Thyroid transcription factor 1

## Competing interests

The authors declare that they have no competing interests.

## Authors' contributions

SRF*, IA* and AJA performed the experiments and helped revising the manuscript; OB participated in the conception and design of the study and helped revise the manuscript; TG and MKM participated in the conception, design and coordination of the study, and drafted the manuscript. All authors read and approved the final manuscript.

*These authors contributed equally to the project and should both be considered first authors.

## Supplementary Material

Additional file 1 **Passing cells through single cell filters before seeding does not affect branching behavior**. The figure displays the colony growth and extent of branching in cultures where cells were seeded after passing through a single cell filter in comparison to unfiltered single cell suspensions. Click here for file

Additional file 2 **Distribution of epithelial markers at different stages of colony development**. The figure shows the overall distribution of epithelial markers in the different colony categories; spherical, budding, early branching and complex branching. Click here for file

Additional file 3 **Inhibition of FGFR signaling affects colony growth**. The figure shows the effect of FGFR inhibition with SU5402 on colony growth when the inhibitor is present from the time of seeding. Click here for file
