# Supplementary material for: Airway branching morphogenesis in three dimensional culture
Source: Respir Res. 2010 Nov 25;11(1):162. doi: 10.1186/1465-9921-11-162 (PMC3002372; doi:10.1186/1465-9921-11-162)
Supplement: Additional file 1 — Passing cells through single cell filters before seeding does not affect branching behavior. The figure displays the colony growth and extent of branching in cultures where cells were seeded after passing through a single cell filter in comparison to unfiltered single cell suspensions. [file 1465-9921-11-162-S1.PDF]

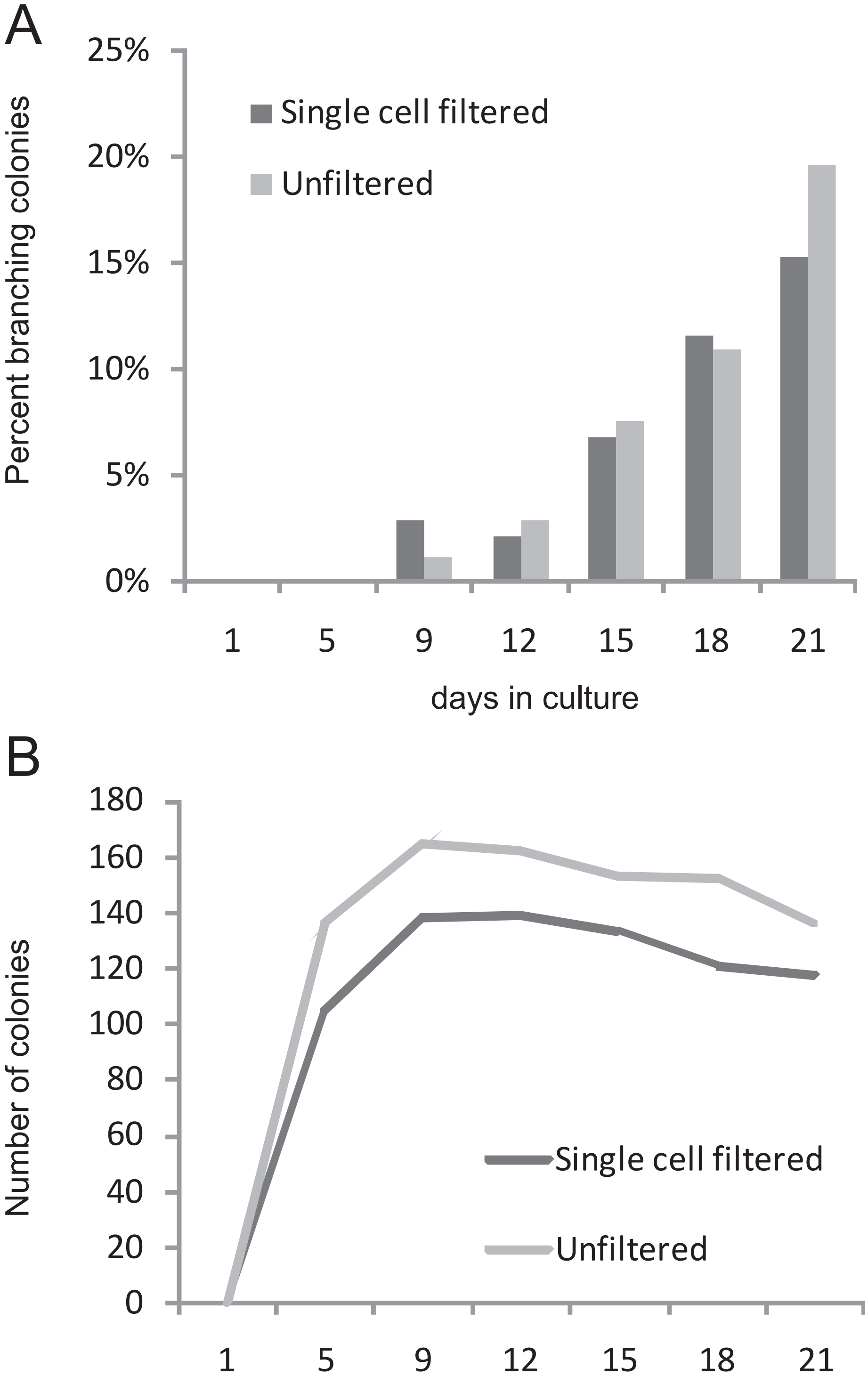

Additional file 1. Passing cells through single cell filters before seeding does not affect branching behavior.

A. The percentage of branching cells over time in cultures where cells were first passed through a 30μm single cell strainer (dark bars) and unfiltered cultures (light bars). **B.** The total number of colonies in the same cultures. Less colonies are formed after filtering cells, indicating that aggregation may play a role in the initial formation of up to 15% of colonies, but not in the branching capability of these colonies.
