# Supplementary material for: Airway branching morphogenesis in three dimensional culture
Source: Respir Res. 2010 Nov 25;11(1):162. doi: 10.1186/1465-9921-11-162 (PMC3002372; doi:10.1186/1465-9921-11-162)
Supplement: Additional file 2 — Distribution of epithelial markers at different stages of colony development. The figure shows the overall distribution of epithelial markers in the different colony categories; spherical, budding, early branching and complex branching. [file 1465-9921-11-162-S2.PDF]

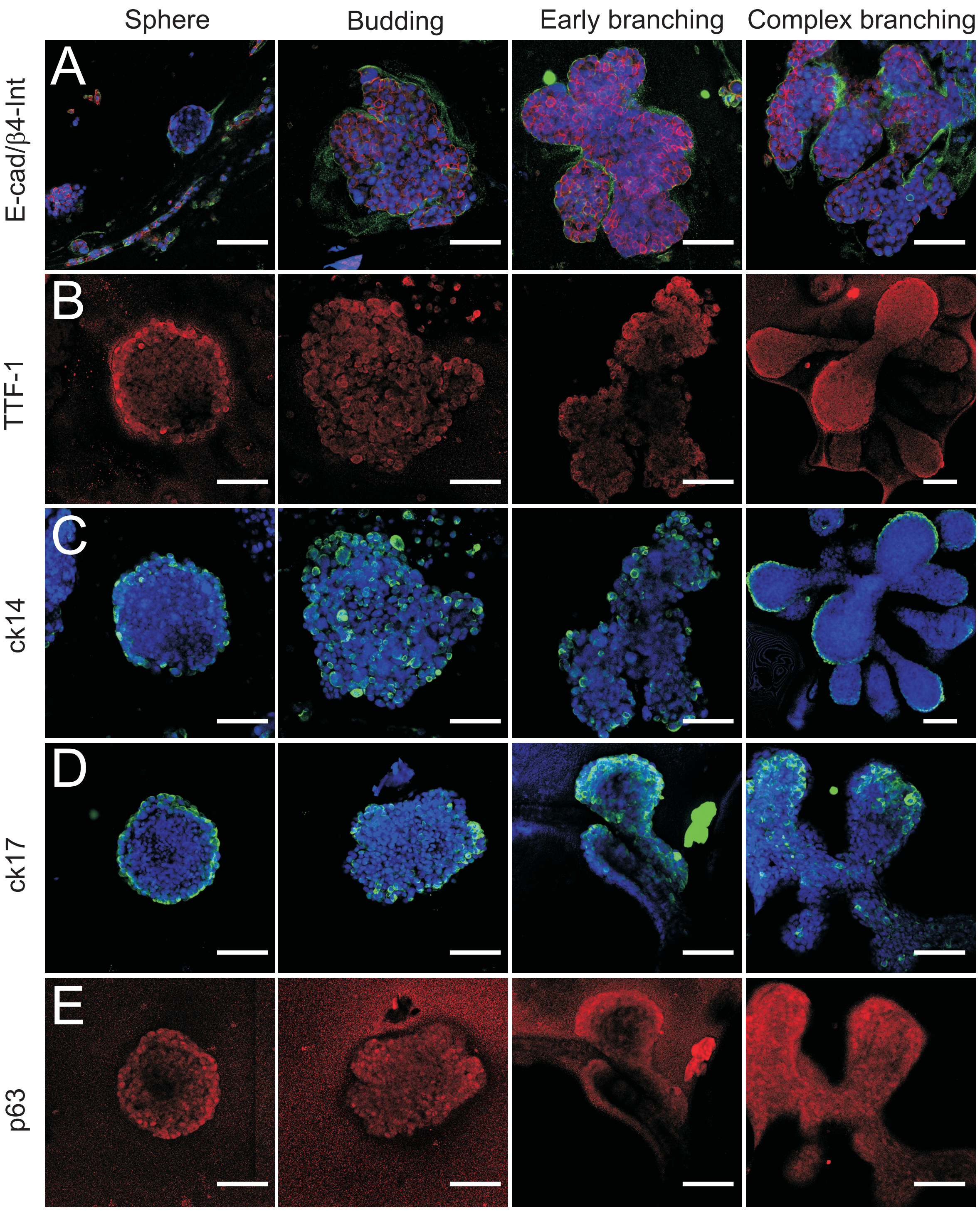

Additional file 2. Distribution of epithelial markers at different stages of colony development. See legend on next page.

Additional file 2. Distribution of epithelial markers at different stages of colony development.

Confocal sections through the core of each structure. Nuclear staining with TO-PRO-3 in blue. The colony type is indicated at the top. **A.** E-cadherin in red, Beta4-Integrin in green. E-cadherin is relatively evenly distributed throughout structures, although small areas showing no staining can be found at the core of some structures. **B.** TTF-1 (red) is found throughout structures but the expression is stronger at the edges of all types of structures. **C.** Ck14 (green) is expressed marginally in spheres and complex or mature branching structures (far left and far right, respectively). In structures undergoing budding and branching, expression appears random in a subset of cells distributed throughout the structure. **D.** Ck17 (green) is more widely expressed than ck14 but the expression remains stronger near the edges at all stages. **E.** p63 staining is seen in all cells, but is stronger in the outermost cells at all stages. B+C and D+E show stainings of the same structures. Scale bars 100 μm.
