# Supplementary material for: Airway branching morphogenesis in three dimensional culture
Source: Respir Res. 2010 Nov 25;11(1):162. doi: 10.1186/1465-9921-11-162 (PMC3002372; doi:10.1186/1465-9921-11-162)
Supplement: Additional file 3 — Inhibition of FGFR signaling affects colony growth. The figure shows the effect of FGFR inhibition with SU5402 on colony growth when the inhibitor is present from the time of seeding. [file 1465-9921-11-162-S3.PDF]

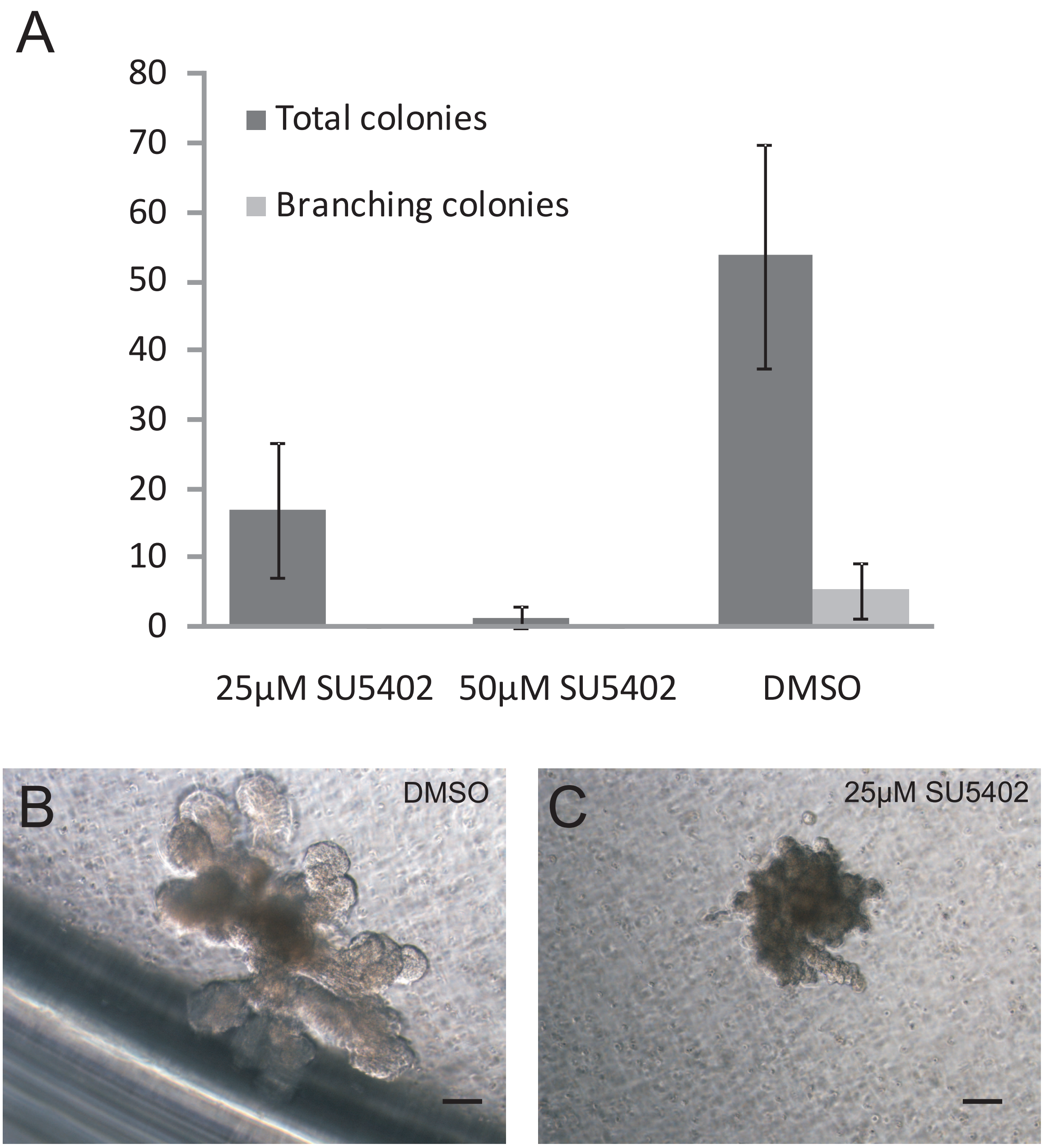

Additional file 3. Inhibition of FGFR signaling affects colony growth.

A. Total number of colonies (dark bars) and branching colonies (light bars) at day 19 after seeding. SU5402 or DMSO was added to the gel and culture medium upon seeding. Colony formation was strongly reduced with the inhibitor and no branching was observed. Error bars indicate standard deviation. **B.** Example of a branching colony in the DMSO control. **C.** Irregular, grape-like colony characteristic of the 25 µM SU5402 cultures. Scale-bars 100 µm.
